# KDEF-PT: Valence, Emotional Intensity, Familiarity and Attractiveness Ratings of Angry, Neutral, and Happy Faces

**DOI:** 10.3389/fpsyg.2017.02181

**Published:** 2017-12-19

**Authors:** Margarida V. Garrido, Marília Prada

**Affiliations:** Instituto Universitário de Lisboa (ISCTE-IUL), CIS – IUL, Lisboa, Portugal

**Keywords:** facial expressions, normative data, subjective ratings, emotion labeling, sex differences

## Abstract

The Karolinska Directed Emotional Faces (KDEF) is one of the most widely used human facial expressions database. Almost a decade after the original validation study (Goeleven et al., 2008), we present subjective rating norms for a sub-set of 210 pictures which depict 70 models (half female) each displaying an angry, happy and neutral facial expressions. Our main goals were to provide an additional and updated validation to this database, using a sample from a different nationality (*N* = 155 Portuguese students, *M* = 23.73 years old, *SD* = 7.24) and to extend the number of subjective dimensions used to evaluate each image. Specifically, participants reported emotional labeling (forced-choice task) and evaluated the emotional intensity and valence of the expression, as well as the attractiveness and familiarity of the model (7-points rating scales). Overall, results show that happy faces obtained the highest ratings across evaluative dimensions and emotion labeling accuracy. Female (vs. male) models were perceived as more attractive, familiar and positive. The sex of the model also moderated the accuracy of emotional labeling and ratings of different facial expressions. Each picture of the set was categorized as low, moderate, or high for each dimension. Normative data for each stimulus (hits proportion, means, standard deviations, and confidence intervals per evaluative dimension) is available as supplementary material (available at https://osf.io/fvc4m/).

## Introduction

The human face conveys important information for social interaction. For example, it is a major source for forming first impressions, and to make fast and automatic personality trait inferences (for a review, see Zebrowitz, 2017). Indeed, facial expressions have been the most studied non-verbal emotional cue (for a review, see Schirmer and Adolphs, 2017). In addition to their physical component (i.e., morphological changes in the face such as frowning or opening the mouth), emotional facial expressions also have an affective component that conveys information about the internal feelings of the person expressing it (for a review, see Calvo and Nummenmaa, 2016). Moreover, facial expressions communicate a social message that informs about the behavioral intentions of the expresser, which in turn prompt responses in the perceiver such approach and avoidance reactions (for a review, see Paulus and Wentura, 2016).

According to a recent meta-analysis, static human faces are the most used stimuli to investigate facial emotion recognition (Paiva-Silva et al., 2016). Not surprisingly, the number of validated image databases depicting facial expressions currently available in the literature is extensive (for reviews, see Bänziger et al., 2012; Kaulard et al., 2012).

These databases are varied regarding the characteristics of the stimuli they comprise as well as the procedures used to validate them (for a review, see Garrido et al., 2016). For example, databases are heterogeneous regarding the characteristics of the models (e.g., age, ethnicity, nationality, amateur volunteers or professional actors) or the type of expressions depicted (e.g., specific emotions, mental states, etc.). In most validation procedures, participants are only asked to categorize the emotion displayed in the face presented by selecting the corresponding emotion label (i.e., forced-choice task). Remarkably, the number of databases that assess other evaluative dimensions is quite limited. The Radboud Faces Database (RaFD – Langner et al., 2010) constitutes an important exception given that it also includes measures of overall valence and target attractiveness and ratings of the intensity, clarity and genuineness of the expression. Also, the Chicago Face Database (CFD – Ma et al., 2015) includes a set of subjective ratings (e.g., attractive, baby-faced, unusual) along with target categorization measures (age estimation, racial/ethnic categorization, gender identification). Another recent example is the Stills And Videos of facial Expressions (SAVE – Garrido et al., 2016) that presents overall ratings of the model (i.e., attractiveness, familiarity and similarity) as well as ratings of the emotional expression (arousal, clarity, genuineness, intensity, valence).

In the current work we will focus on the Karolinska Directed Emotional Faces (KDEF), an image set developed by Lundqvist et al. (1998). The full set comprises 4900 standardized pictures of human facial expressions portrayed by 70 models (white Swedish amateur actors with ages between 20 and 30 years old, half of them were women). Each model was photographed twice (Series A and B) displaying seven emotional expressions (angry, fearful, disgusted, sad, happy, surprised, and neutral) from five different angles (full left profile, half left profile, straight, half right profile, full right profile). The models were instructed to evoke each emotion and to display it strongly and clearly. Besides age, selection criteria included the absence of facial hair, earrings or eyeglasses, and visible make-up during the photo-session.

A validation study for a subset of these pictures was subsequently published by Goeleven et al. (2008). Specifically, this subset comprised 490 frontal view pictures (Series A) of the 70 models displaying all seven emotional expressions. For the validation study, the models’ hair line was removed to “minimize fashion issues” (p. 1096) and to “make the facial emotion expression clearer” (Goeleven et al., 2008, p. 1102). Participants (students from a Belgian University) were asked to perform an emotion recognition task as well as to provide ratings of intensity (9-point rating scale) and arousal (graphic 9-point scale – Self-Assessment Manikin; Lang, 1980). Overall, based on the hit rates analysis and the test–retest results, this KDEF subset offers a valid set of pictorial affective faces.

The KDEF validation study (Goeleven et al., 2008) has been consistently used and cited numerous times (182 Web of Science; 294 Google Scholar, search conducted on June 27, 2017) throughout the (almost) 10 years following its publication. Indeed, the KDEF pictures have been used as materials in several research domains. For example, these pictures were used to investigate the role played by contextual factors (categorization goals) in shaping responses to facial expressions, using both behavioral (response times) and psychophysiological (EMG) measures (van Dillen et al., 2015). The KDEF was also used to investigate how individual (e.g., sex of the rater – Hong et al., 2015) or cultural (e.g., Zhang et al., 2015) variables modulate emotion recognition. The KDEF pictures (happy and neutral) have also been used to examine the influence of smiling on the age estimation of the models (Ganel, 2015, Experiment 1a).

Emotional faces, including those in the KDEF database, are commonly used in priming studies focusing on affective processing (e.g., Wentura et al., 2017). For example, KDEF pictures were used as prime stimuli in a recent study examining the influence of emotional faces on food processing (Manippa et al., 2017). Other applications include studies aimed at examining the impact of specific emotional expressions on persuasion outcomes (Van Kleef et al., 2015; Calanchini et al., 2016).

Despite the extensive use of KDEF, to our knowledge, and aside from the work by Goeleven et al. (2008), there are only two other validation studies focusing exclusively on this database. First, Calvo and Lundqvist (2008) presented normative ratings of a set of 280 frontal pictures (40 models, half female) depicting all available facial expressions (i.e., angry, fearful, disgusted, happy, sad, surprised, and neutral). Stimuli were presented in fixed durations (25, 50, 100, 250, and 500 ms) or in the absence of a time limit (“free-viewing condition”) and participants (Spanish nationality) were asked to recognize the emotions displayed (forced choice task). The norms include accuracy and response times for each stimulus and facial expression across the different exposure times. Overall, the authors found an advantage for the processing of happy faces that were identified more accurately, and faster than the other expressions. Moreover, although recognition was improved by longer presentation displays for the remaining expressions, in the case of happy pictures a ceiling effect on the accuracy level was observed at 50 ms.

The other normative study was conducted by Sánchez and Vázquez (2013) who validated a sub-set of 198 angry, sad, and happy frontal view KDEF pictures using a distinct procedure (“anchor-point method”). Specifically, each emotional picture was paired with a neutral one of the same model (8 s display) and participants (Spanish nationality) were asked to judge the intensity of each emotional expression, as well as its prototypicality. Overall, results showed that happy faces were perceived as more prototypical than both angry and sad faces. Regarding, intensity, happy and angry faces were judged as more intense than sad ones.

Despite the significant contribution of both of these studies, it is noteworthy that the first (Calvo and Lundqvist, 2008) is limited to emotional recognition norms and that the second (Sánchez and Vázquez, 2013) did not include norms of the neutral faces and used a procedure that compared each emotional expression, regarding its intensity and prototypicality, to the corresponding neutral one. That is not the case in most studies that use facial expressions as stimulus materials.

The fact that the KDEF stimuli are still being extensively used almost a decade after the publication of its original validation (Goeleven et al., 2008) supports the pertinence of conducting a new normative study. This was the goal of the current work: to validate a sub-set of pictures selected from the KDEF database using a sample of a different nationality (Portuguese participants) and extending the number of subjective evaluative dimensions used to assess each stimulus (i.e., emotional intensity and valence of the expression, and attractiveness and familiarity of the model). The validity of stimuli databases may not be guaranteed when used with participants from a different culture. Indeed, cross-national validation is a frequent procedure for other visual stimuli databases. For example, the original normative study of International Affective Picture System (Lang et al., 2008) conducted with North American (United States) participants was subsequently adapted to multiple countries/cultures (e.g., China, India, Belgium, Portugal, for a review, see Soares et al., 2015). Also, the Food-pics database (Blechert et al., 2014) was originally validated using German-speaking (including participants from Germany, Switzerland, and Austria) and United States samples and was recently validated with a Portuguese sample (Prada et al., 2017). Cross-cultural validation of databases of facial expressions is particularly advised for a number of reasons. For instance, research has suggested that both the experience and display of emotion (for reviews, see Immordino-Yang and Yang, 2017; Niedenthal et al., 2017), as well as emotional recognition (for reviews, see Chen and Jack, 2017; Gendron, 2017) may vary across cultures. Several studies have shown an advantage in emotion recognition when the targets are members of the in-group (e.g., Yan et al., 2017; for a review, see Elfenbein, 2015). For example, Yan et al. (2016) showed that, despite the considerable cross-cultural agreement regarding the categorization of different expressions, Caucasian participants made more errors when the stimuli depicted Chinese (vs. Caucasian – KDEF database) models, whereas Chinese participants showed the reverse pattern. Likewise, in the validation study of the Amsterdam Dynamic Facial Expression Set, van der Schalk et al. (2011) reported that Dutch participants were generally more accurate in recognizing emotional displays of Northern European models than Mediterranean models.

The normative data is particularly useful for the Portuguese research community, as it provides access to ready-to-use materials. Nevertheless, the applicability of our work is not limited to Portugal because we have extended the number of subjective dimensions assessed, increasing the scope of KDEF. The current study comprises the entire set of 70 models (half female). However, only pictures displaying a negative (anger), neutral, and positive (happiness) facial expression were included. Angry and happy expressions were selected because they are deemed to be of opposite valence, to implicate distinct facial muscles and to produce different emotional responses (for a review, see Cañadas et al., 2016). Emotional faces assume a communicative and adaptive value that have been shown to influence attentional processing and remembering (e.g., angry faces have been shown to be more resistant to forgetting than happy faces – Tay and Yang, 2017). Also, a set of experiments focusing on the social functions of emotional expressions, concluded that angry (vs. happy) expressions were more strongly associated with rejection (vs. acceptance) than other facial expressions (Heerdink et al., 2015). Finally, happy and angry expressions are typically associated with high recognition rates (e.g., Goeleven et al., 2008; Langner et al., 2010).

Previous research has also suggested that the sex of the expresser plays a role in the perception of emotional faces (e.g., Adolph and Alpers, 2010). For example, Becker et al. (2007) demonstrated an advantage (i.e., higher accuracy, lower response times) in the detection of angry expressions on male faces and of happy expressions on female faces. Likewise, Tay and Yang (2017) showed better recognition and recall for angry expressions on male faces and happy expressions on female faces.

Former studies used angry and happy faces to activate valence (e.g., Murphy and Zajonc, 1993) or, more specifically, as exemplars of socially aversive versus appetitive stimuli (e.g., Enter et al., 2014). Angry faces, for instance, are defined as threatening stimuli, being particularly useful to study how the processing of facial affect differs in certain clinical populations (e.g., individuals with social anxiety disorder – Jusyte and Schönenberg, 2014).

Obtaining norms relative to neutral facial expressions is also highly relevant because these stimuli may be used as baseline in a myriad of paradigms, such as affective priming (e.g., Dimberg et al., 2000; Winkielman et al., 2005) or approach-avoidance tasks (e.g., Heuer et al., 2007; Enter et al., 2014). Neutral faces have also been used as exemplars of ambiguous stimuli that may be interpreted according to individual variables of the perceiver (e.g., Farc et al., 2008) or their knowledge about the target (e.g., Suess et al., 2014). Neutral faces are also useful for studies in the perception domain. For example, they have been used as target-stimuli in impression formation tasks that require participants to judge the personality of the target rather than a given emotional state (Bar et al., 2006). However, the literature on emotion recognition has overlooked the accuracy in neutral face recognition (for a discussion on this matter, see Lewinski, 2015) which emphasizes the importance of including this kind of stimuli in validation studies.

Besides emotion recognition, in the current study, each image was evaluated regarding additional model characteristics (attractiveness and familiarity), and features of the facial expression (valence and emotional intensity). Intensity of the expression facilitates emotion recognition (e.g., Adolph and Alpers, 2010). Moreover, previous research has suggested that these evaluative dimensions are interrelated and may vary according to the facial expression (for a review, see Garrido et al., 2016). For example, the same model is perceived as more attractive when displaying a happy expression than when displaying a sad (Mueser et al., 1984; Ueda et al., 2016) or angry (Morrison et al., 2013) expression. Indeed, the relationship between attractiveness and happiness seems to be bidirectional – the ratings of attractiveness are influenced by the intensity of the smile and attractive faces are more easily recognized as happy (Golle et al., 2014). It is noteworthy that the intensity of positive and negative expressions asymmetrically influences attractiveness evaluations, such that faces with more intense happy expressions are deemed more attractive, whereas no significant relationship between attractiveness and intensity emerges for sad expressions (Ueda et al., 2016). Familiarity also influences the perception of facial expressions. For example, a recent study showed that that faces of individuals who had previously been shown (i.e., repeated exposure) were deemed happier than novel faces (Carr et al., 2017; see also, Claypool et al., 2007).

## Materials and Methods

### Participants and Design

The sample included 155 university students (83.20% female; *M*_age_ = 23.73; *SD* = 7.24), from two universities in Lisbon, who volunteered to participate in the present study^1^. The design included two within-participants factors: 2 (sex of the model: male; female) × 3 (facial expression: angry; neutral; happy).

### Materials

Our stimuli set comprised 210 pictures of human facial expressions with no hairline selected from the KDEF original validation (Goeleven et al., 2008), which included 490 frontal view pictures of 70 models (35 female), each displaying six basic emotions (anger, fear, disgust, happiness, sadness, and surprise) and a neutral facial expression. For the present study, we selected all the pictures depicting anger, happiness and neutral facial expressions for each of the 70 models. The pictures had 3.5 cm × 3.5 cm (562 pixels × 562 pixels). These measures are based on the biometric passport photo standards in use around the world. The pictures were printed in a high-quality laser printer in gray scale.

### Procedure

All the procedures were conducted in line with the ethical guidelines of the host institution (ISCTE-IUL, CIS – IUL), including verbal informed consent from all subjects. Participants were invited to collaborate in a validation study of emotional pictures of human faces to be used in future research. The experimenter informed the participants about the goals of the study (i.e., evaluation of faces in multiple dimensions), its expected duration (approximately 20 min), and about ethical considerations (i.e., voluntary nature of the participation, anonymity, confidentiality and the possibility to withdraw from the study at any point). Data were collected in group sessions.

After agreeing to participate, participants were further informed that what the task required was something that people frequently and easily do in everyday life and that judgments should be based on first impressions. Each participant received a booklet with 54 or 51 randomly selected pictures of facial expressions (six per page). Participants were asked to rate each picture on four evaluative dimensions (valence, emotional intensity, familiarity and attractiveness), using 7-point rating scales, and to label the emotion displayed. The booklet included an initial section explaining the meaning of these dimensions as well as instructions stating that the ratings should be based on their first impression on the picture. Specifically, participants were asked to rate the valence and the emotional intensity of the facial expression portrayed (1 = *Very negative* to 7 = *Very Positive* and 1 = *Not at all intense* to 7 = *Very Intense*, respectively). Participants were also asked to indicate the extent to which the model looked familiar and attractive (1 = *Not at all familiar* to 7 = *Very Familiar* and 1 = *Not at all attractive* to 7 = *Very attractive*, respectively). Regarding emotion labeling, participants were instructed to selected the word that best described the emotion displaying by the model – “angry,” “neutral,” “happy,” or “other.”

In order to prevent fatigue and demotivation, each participant evaluated a sub-set of pictures. The stimuli were distributed by four lists: two of the lists included 18 models displaying the three facial expressions (happy, neutral, angry, 54 pictures per list), and the other two included 17 models displaying the three facial expressions (51 pictures per list). The distribution of models per list was random, but the sex of the model was counterbalanced. Each list was presented in four versions, with pictures ordered based on a list of random numbers (total of 16 versions of the booklets). Participants took approximately 20 min to fill in the questionnaire.

## Results

### Statistical Analysis

Each picture was rated by a minimum of 33 and a maximum of 42 participants. The analysis of the responses to the subjective evaluative dimensions, shows a low percentage of both missing cases and outliers (0.54 and 1.16%, respectively). Outliers were identified considering the criterion of 2.5 standard deviations above or below the mean evaluation of each stimulus in a given dimension. There was also no indication of participants responding systematically in the same way that is, always using the same value of the scale. Therefore, no further participants were excluded.

In the following sections, we analyze the impact of facial expression (i.e., angry, neutral, happy), and of the sex of the model (e.g., Calvo and Lundqvist, 2008), on the accuracy of emotion labeling (see Emotion Labeling Task) and ratings on each subjective dimension (i.e., attractiveness, familiarity, intensity, and valence, see Impact of Facial Expression and Model’s Sex on Evaluative Dimensions). Specifically, for each dependent variable we conducted a repeated measures ANOVA, with facial expression and sex of the model defined as within-participants factors. Given the high prevalence of female participants in the sample, we repeated these analyses weighting the cases to reflect female and male effectives in the Portuguese population (i.e., weighting factors: Female = 0.62; Male = 2.92). The few discrepancies in the data pattern resulting from these analyzes are presented in the respective section.

We also examine the associations between evaluative dimensions (see Associations Between Dimensions) and present a general characterization of the stimuli set according to the frequency of pictures categorized as low, moderate or high on each dimension (see Frequency Distribution). Moreover, we present item-level data including the proportion of hits in the emotion labeling task and descriptive statistics (means, standard deviations, and confidence intervals) for each evaluative dimension. These normative ratings are freely available as supplementary material at https://osf.io/fvc4m/.

### Emotion Labeling Task

The mean proportion of hits (i.e., correct categorization of the facial expression) was calculated, per participant, according to the expression displayed and the sex of the model. The overall mean proportion of hits was 0.74 (*SD* = 0.14). We observed a main effect of facial expression on hits proportion, *F*(2,308) = 71.75, *MSE* = 5.48, *p* < 0.001, $\eta_{p}^{2}$ = 0.318, such that the hits proportion of pictures portraying happy expressions (*M* = 0.89, *SD* = 0.14) was higher than pictures portraying either angry (*M* = 0.69, *SD* = 0.19), *t*(154) = 12.83, *p* < 0.001, *d* = 1.03, or neutral expressions (*M* = 0.64, *SD* = 0.29), *t*(154) = 11.23, *p* < 0.001, *d* = 0.90. The difference between the proportion of hits of neutral and angry expressions was not significant, *t*(154) = -1.89, *p* = 0.061, *d* = 0.15.

Although the main effect of model’s sex on hits proportion was not significant, *F*(1,154) = 3.15, *MSE* = 0.71, *p* = 0.078, $\eta_{p}^{2}$ = 0.020, results show an interaction between model’s sex and facial expression recognition, *F*(2,308) = 13.08, *MSE* = 0.22, *p* < 0.001, $\eta_{p}^{2}$ = 0.078. Specifically, when the pictures displayed angry expressions, the hits proportion was higher for male models (*M* = 0.72, *SD* = 0.22) than for female models (*M* = 0.65, *SD* = 0.22), *t*(154) = 3.91, *p* < 0.001, *d* = 0.31. The reverse was true for pictures displaying happy expressions, that is, higher hits proportion for female (*M* = 0.91, *SD* = 0.14) than for male models (*M* = 0.87, *SD* = 0.17), *t*(154) = -3.17, *p* < 0.001, *d* = 0.25. No differences in hits proportion were found regarding pictures depicting neutral expressions for female (*M* = 0.63, *SD* = 0.31) and male models (*M* = 0.65, *SD* = 0.30), *t*(154) = -1.33, *p* = 0.186, *d* = 0.11.

### Impact of Facial Expression and Model’s Sex on Evaluative Dimensions

The evaluation of each target was examined by computing the mean ratings, per participant, in each dimension for the three types of facial expression (angry, neutral, and happy) according to the sex of the model. Results are summarized in **Table 1**.

**Table 1:** Evaluations (mean and standard deviation) in each dimension as a function of model’s sex and facial expression.

	Angry		Neutral		Happy		Total	
	*M*	*SD*	*M*	*SD*	*M*	*SD*	*M*	(*SD*)
**Attractiveness**					
Female	2.35^a^	(0.80)	3.13^b^	(0.92)	4.29^c^	(1.25)	3.26^1^	(0.81)
Male	2.10^a^	(0.74)	2.98^b^	(0.88)	3.84^c^	(1.27)	2.97^2^	(0.83)
Total	2.23^d^	(0.73)	3.06^e^	(0.86)	4.07^f^	(1.21)		
**Familiarity**					
Female	3.24^a^	(1.27)	3.59^b^	(1.24)	4.41^c^	(1.50)	3.75^1^	(1.17)
Male	3.07^a^	(1.23)	3.55^b^	(1.20)	4.26^c^	(1.45)	3.62^2^	(1.15)
Total	3.15^d^	(1.23)	3.57^e^	(1.19)	4.33^f^	(1.43)		
**Intensity**					
Female	5.00^a^	(0.89)	3.44^b^	(0.89)	5.02^a^	(1.05)	4.49^1^	(0.72)
Male	4.99^a^	(0.90)	3.48^b^	(0.85)	4.82^c^	(0.98)	4.43^1^	(0.67)
Total	5.00^d^	(0.83)	3.46^e^	(0.84)	4.92^d^	(0.97)		
**Valence**					
Female	2.59^a^	(0.65)	3.69^b^	(0.43)	5.74^c^	(0.73)	4.01^1^	(0.36)
Male	2.47^a^	(0.62)	3.68^b^	(0.40)	5.54^c^	(0.74)	3.90^2^	(0.34)
Total	2.53^d^	(0.58)	3.69^e^	(0.37)	5.64^f^	(0.69)		

*Means in the same line that share the same superscript – ^a,b,c^ (means associated with the interaction between facial expression and model’s sex) and – ^d,e,f^ (means associated with the main effect of facial expression) – did not differ significantly. Means in the same column that share the same superscript – ^1,2^ (means associated to the main effect of model’s sex) – did not differ significantly. Pairwise comparisons with Bonferroni correction.*

#### Attractiveness

We found a main effect of facial expression on attractiveness ratings, *F*(2,308) = 307.39, *MSE* = 261.75, *p* < 0.001, $\eta_{p}^{2}$ = 0.666, with models portraying a happy expression evaluated as the most attractive, followed by those with a neutral or angry expression. There was also a main effect of model’s sex on this dimension, *F*(1,154) = 63.69, *MSE* = 18.70, *p* < 0.001, $\eta_{p}^{2}$ = 0.087, such that female models were always evaluated as more attractive than male models. The interaction between factors was also significant, *F*(2,308) = 15.77, *MSE* = 1.85, *p* < 0.001, $\eta_{p}^{2}$ = 0.093. Specifically, the interaction shows that, although female participants were always rated as more attractive than male models, the difference in attractiveness is stronger when the models are displaying a happy expression.

#### Familiarity

We found a main effect of facial expression on familiarity ratings, *F*(2,308) = 112.09, *MSE* = 111.30, *p* < 0.001, $\eta_{p}^{2}$ = 0.421, with models displaying a happy expression evaluated as the most familiar, followed by those with a neutral or angry expression. There was also a main effect of model’s sex on this dimension, *F*(1,154) = 19.71, *MSE* = 3.53, *p* < 0.001, $\eta_{p}^{2}$ = 0.113, such that female models were always evaluated as more familiar than male models. The interaction between factors was not significant, *F*(2,308) = 1.93, *MSE* = 0.34, *p* = 0.147, $\eta_{p}^{2}$ = 0.012.^2^

#### Emotional Intensity

We found a main effect of facial expression on emotional intensity ratings, *F*(2,308) = 234.25, *MSE* = 233.18, *p* < 0.001, $\eta_{p}^{2}$ = 0.603, such that models displaying either an angry or a happy expression obtained higher intensity ratings than those displaying a neutral expression. There was no main effect of model’s sex on this dimension, *F*(1,154) = 3.59, *MSE* = 0.72, *p* = 0.060, $\eta_{p}^{2}$ = 0.023.^3^ However, results show an interaction between this factor and facial expression *F*(2,308) = 8.37, *MSE* = 1.32, *p* < 0.001, $\eta_{p}^{2}$ = 0.052. Specifically, pictures depicting female models displaying a happy expression were rated as more intense than pictures depicting male models with the same expression. No differences according to the sex of the model were observed for angry and neutral expressions.

#### Valence

We found a main effect of facial expression on valence ratings, *F*(2,308) = 1190.78, *MSE* = 764.94, *p* < 0.001, $\eta_{p}^{2}$ = 0.885, such that pictures depicting happy expressions were rated as the most positive, followed by those depicting neutral or angry expressions. There was also a main effect of model’s sex on this dimension, *F*(1,154) = 22.43, *MSE* = 2.92, *p* < 0.001, $\eta_{p}^{2}$ = 0.127, such that female models were evaluated more positively that male models. The interaction between factors, *F*(2,308) = 7.27, *MSE* = 0.71, *p* = 0.001, $\eta_{p}^{2}$ = 0.045, shows that pictures with female models displaying either angry or happy expressions were perceived as more positive than pictures with male models, whereas no differences were found according to model’s sex for pictures depicting neutral facial expressions.

### Associations between Dimensions

We observed that all the evaluative dimensions were positively correlated. Due to the high number of ratings, all the correlations were statistically significant (all *p*s < 0.001). Specifically, we observed a strong positive correlation between attractiveness and both valence (*r* = 0.56), and familiarity (*r* = 0.52). The correlation between attractiveness and emotional intensity ratings was also positive (*r* = 0.17), although not as strong. Familiarity was also positively correlated with valence (*r* = 0.34) and with emotional intensity (*r* = 0.18). Finally, the association between the latter two dimensions was also positive, but weaker than the remaining (*r* = 0.09).

### Frequency Distribution

We computed means, standard deviations and confidence intervals (CI) for each stimulus in each evaluative dimension (see Supplementary Material available at https://osf.io/fvc4m/). Based on the confidence interval, pictures were categorized as low, moderate or high on each dimension. When the CI included the response scale midpoint (i.e., 4) the pictures were categorized as moderate. When the upper bound of the CI was below the scale midpoint, the pictures were categorized as low, whereas when the lower bound of the CI was above the scale midpoint, they were categorized as high (for similar procedure, see Prada et al., 2016; Rodrigues et al., 2017). These results are summarized in **Table 2**.

**Table 2:** Distribution of pictures (%) across each dimension level as a function of facial expression.

	Overall			Angry			Neutral			Happy		
	L	M	H	L	M	H	L	M	H	L	M	H
Attractiveness	66.7	25.7	7.6	100.0	–	–	85.7	14.3	–	14.3	62.9	22.9
Familiarity	41.0	50.0	9.0	88.6	11.4	–	32.9	67.1	–	1.4	71.4	27.1
Emotional intensity	25.7	19.0	55.2	–	17.1	82.9	75.7	24.3	–	1.4	15.7	82.9
Valence	52.9	13.8	33.3	97.1	2.9	–	61.4	37.1	1.4	–	1.4	98.6

*L = Low, M = Moderate, H = High. For valence: Low = Negative, Moderate = Neutral, High = Positive.*

Results showed that, overall, the models were perceived as low in attractiveness (66.7%) and moderate in familiarity (50.5%). Also, approximately half (52.9%) of the facial expressions portrayed were categorized as negative and as high in emotional intensity (55.22%). Pictures depicting angry facial expressions were perceived as low in attractiveness, and mostly as negative, low in familiarity and high in emotional intensity. Pictures depicting neutral facial expressions were mostly perceived as negative, moderate in familiarity and as low in attractiveness and emotional intensity. Finally, pictures depicting happy facial expressions were perceived as positive, moderate in attractiveness and familiarity, and as high in emotional intensity.

## Discussion

This study presents subjective rating norms of 210 pictures of angry, neutral, and happy facial expressions selected from the KDEF database. A Portuguese sample categorized the emotion conveyed by each stimulus and rated its valence and emotional intensity. Moreover, each image was also rated regarding the attractiveness and familiarity of the model. Overall, we observed positive correlations between these variables, in particular between attractiveness and both valence and familiarity. Our findings showed that the current stimuli set is varied across evaluative dimensions and yielded emotional accuracy rates similar to the original KDEF study (Goeleven et al., 2008), suggesting its suitability for research conducted in other countries.

We observed an advantage of happy (vs. angry and neutral) faces in emotion labeling, which is in line with the literature, namely with the original KDEF validation. For example a review by Nelson and Russell (2013), concluded that happiness is the emotion with the highest percentage of hits (around 90%) across cultures and languages. Indeed, the identification threshold for a happy expression is particularly low, given that this emotion can be recognized even when presented very fast (e.g., Calvo and Lundqvist, 2008) and with minimal intensity (Calvo et al., 2016). Moreover, happy faces not only were perceived as the most positive and familiar (e.g., Garrido et al., 2016), but also as the most attractive (e.g., Golle et al., 2014; Garrido et al., 2016). Regarding emotional intensity, happy and angry faces were perceived as more intense than neutral faces (e.g., Garrido et al., 2016).

The hit rates for the emotion labeling of neutral faces were also similar to those reported in the original validation (Goeleven et al., 2008). However, for angry faces we found lower hit rates than those reported by Goeleven et al. (2008).

Previous studies have suggested that the sex of the expresser is a factor to be taken into account when working with pictures of facial expressions (Adolph and Alpers, 2010). In Calvo and Lundqvist’s (2008) KDEF validation study, no systematic effects of model sex were reported for emotion labeling. In contrast, we observed that participants were more accurate in identifying the expression of anger in male (vs. female) faces and in identifying the expression of happiness in female (vs. male) faces (see also, Becker et al., 2007). Importantly, such model sex differences were not found for neutral faces. Moreover, we also found effects of model sex on attractiveness, familiarity and valence ratings, such that pictures displaying female models obtained higher ratings in such dimensions, irrespectively, of facial expression. It is possible that such effects are due to the fact that the majority of the participants in the current study were also women (e.g., Langner et al., 2010). However, that was also the case in the validation of the SAVE database (Garrido et al., 2016) that only identified an effect of the sex of the model for the attractiveness dimension (i.e., higher attractiveness ratings for female models). This overall pattern of results is replicated when weighting the cases to follow the female and male effectives in the population. However, we acknowledge that the high prevalence of female participants does not allow a proper examination of how the (mis)match between the sex of the participant and of the model impacts the evaluation of facial expressions. This was also a limitation in the original validation that exclusively included female participants. Future studies should include more balanced samples regarding the sex of the participants.

This study extends the norms available for the KDEF pictures because it assesses additional evaluative dimensions, namely valence, attractiveness and familiarity using a sample from a different nationality. Note, however, that the procedure used in the current work differs from the one used in the original validation (Goeleven et al., 2008) in several aspects. For example, we presented the pictures in gray scale and not in color, multiple pictures were presented in a single page (vs. projection of a single picture) and the exposure (and rating) time to each picture was controlled by the participants (vs. fixed duration of 5 s per picture). The fact that we did not impose time restrictions to the evaluation of the stimuli also does not permit to ascertain that the ratings here reported reflect participants’ first impressions (for similar procedure, see Langner et al., 2010; Ma et al., 2015). As in the original KDEF validation, all participants in the current study were students. Although this constitutes a potential limitation to the generalization of the norms, students are often recruited as participants in studies using this type of stimuli.

The validation of pictures of facial expressions is useful for a variety of research areas. For instance, these stimuli can be used to examine differences in emotion perception in clinical versus normative populations. As an illustration, it has been shown that individuals who are depressed are more (vs. less) sensitive to negative (vs. positive) affect cues and more likely to interpret ambiguous emotional stimuli negatively (for a review, see Penton-Voak et al., 2017). Pictures of angry, neutral, and happy expressions can also be used in multiple paradigms. For example, these pictures can be used as primes in affective priming tasks (e.g., Murphy and Zajonc, 1993; Dimberg et al., 2000; Winkielman et al., 2005) and in the Affect Misattribution Procedure (e.g., Heerdink et al., 2015; Rohr et al., 2015).

In sum, the current work updates and extends the norms available for angry, neutral, and happy pictures of one of the most widely used databases of human facial expressions – the KDEF – using participants from a different nationality (i.e., Portuguese) than that of the models used in the development of the set (Swedish models) and that of the samples used in previous validation studies (i.e., Spanish). Moreover, the addition of new subjective evaluative dimensions allows researchers to select adequate stimuli for a given experiment based on several criteria (e.g., manipulate the dimensions of interest) while controlling for others. This database provides a valuable tool of ready-to-use materials that can be applied in several paradigms and research domains.

## Database Access

The database can be found at https://osf.io/fvc4m/.

## Ethics Statement

The research was conducted in compliance with all APA Ethical Guidelines for the treatment of human participants. This study was carried out in accordance with the recommendations of the Code of Ethical Conduct in Research of ISCTE-IUL with verbal informed consent from all subjects.

## Author Contributions

MVG designed the study, created the surveys and supervised data collection. MVG and MP analyzed the data and wrote the manuscript.

## Conflict of Interest Statement

The authors declare that the research was conducted in the absence of any commercial or financial relationships that could be construed as a potential conflict of interest.

## Footnotes

1 Initially the sample included 158 participants. However, three were excluded because they responded to less than 50% of the questionnaire.

2 When the analysis was conducted weighting the cases according to participants’ sex, this interaction becomes significant (i.e., pictures depicting female models rated as more familiar than male models, except for neutral expressions).

3 By weighting the cases according to participants’ sex, this main effect becomes significant (i.e., pictures depicting female models are perceived as more intense).

## References

[B1] Adolph D. Alpers G. W. (2010). Valence and arousal: a comparison of two sets of emotional facial expressions. *Am. J. Psychol.* 123 209–219. 10.5406/amerjpsyc.123.2.0209 20518437

[B2] Bänziger T. Mortillaro M. Scherer K. R. (2012). Introducing the Geneva multimodal expression corpus for experimental research on emotion perception. *Emotion* 12 1161–1179. 10.1037/a0025827 22081890

[B3] Bar M. Neta M. Linz H. (2006). Very first impressions. *Emotion* 6 269–278. 10.1037/1528-3542.6.2.269 16768559

[B4] Becker D. V. Kenrick D. T. Neuberg S. L. Blackwell K. C. Smith D. M. (2007). The confounded nature of angry men and happy women. *J. Pers. Soc. Psychol.* 92 179–190. 10.1037/0022-3514.92.2.179 17279844

[B5] Blechert J. Meule A. Busch N. A. Ohla K. (2014). *Food-pics*: an image database for experimental research on eating and appetite. *Front. Psychol.* 5:617. 10.3389/fpsyg.2014.00617 25009514 PMC4067906

[B6] Calanchini J. Moons W. G. Mackie D. M. (2016). Angry expressions induce extensive processing of persuasive appeals. *J. Exp. Soc. Psychol.* 64 88–98. 10.1016/j.jesp.2016.02.004

[B7] Calvo M. G. Avero P. Fernández-Martín A. Recio G. (2016). Recognition thresholds for static and dynamic emotional faces. *Emotion* 16 1186–1200. 10.1037/emo0000192 27359222

[B8] Calvo M. G. Lundqvist D. (2008). Facial expressions of emotion (KDEF): identification under different display-duration conditions. *Behav. Res. Methods* 40 109–115. 10.3758/BRM.40.1.109 18411533

[B9] Calvo M. G. Nummenmaa L. (2016). Perceptual and affective mechanisms in facial expression recognition: an integrative review. *Cogn. Emot.* 30 1081–1106. 10.1080/02699931.2015.1049124 26212348

[B10] Cañadas E. Lupiáñez J. Kawakami K. Niedenthal P. M. Rodríguez-Bailón R. (2016). Perceiving emotions: cueing social categorization processes and attentional control through facial expressions. *Cogn. Emot.* 30 1149–1163. 10.1080/02699931.2015.1052781 26197208

[B11] Carr E. W. Brady T. F. Winkielman P. (2017). Are you smiling, or have I seen you before? Familiarity makes faces look happier. *Psychol. Sci.* 28 1087–1102. 10.1177/0956797617702003 28594281

[B12] Chen C. Jack R. E. (2017). Discovering cultural differences (and similarities) in facial expressions of emotion. *Curr. Opin. Psychol.* 17 61–66. 10.1016/j.copsyc.2017.06.010 28950974

[B13] Claypool H. M. Hugenberg K. Housley M. K. Mackie D. M. (2007). Familiar eyes are smiling: on the role of familiarity in the perception of facial affect. *Eur. J. Soc. Psychol.* 37 856–866. 10.1002/ejsp.422

[B14] Dimberg U. Thunberg M. Elmehed K. (2000). Unconscious facial reactions to emotional facial expressions. *Psychol. Sci.* 11 86–89. 10.1111/1467-9280.00221 11228851

[B15] Elfenbein H. A. (2015). “In-group advantage and other-group bias in facial emotion recognition,” in *Understanding Facial Expressions in Communication: Cross-Cultural and Multidisciplinary Perspectives*, eds Mandal M. K. Awasthi A. (New Delhi: Springer), 57–71.

[B16] Enter D. Spinhoven P. Roelofs K. (2014). Alleviating social avoidance: effects of single dose testosterone administration on approach–avoidance action. *Horm. Behav.* 65 351–354. 10.1016/j.yhbeh.2014.02.001 24530652

[B17] Farc M.-M. Crouch J. L. Skowronski J. J. Milner J. S. (2008). Hostility ratings by parents at risk for child abuse: impact of chronic and temporary schema activation. *Child Abuse Negl.* 32 177–193. 10.1016/j.chiabu.2007.06.001 18316122

[B18] Ganel T. (2015). Smiling makes you look older. *Psychon. Bull. Rev.* 22 1671–1677. 10.3758/s13423-015-0822-7 25855200

[B19] Garrido M. V. Lopes D. Prada M. Rodrigues D. Jerónimo R. Mourão R. P. (2016). The many faces of a face: comparing stills and videos of facial expressions in eight dimensions (SAVE database). *Behav. Res. Methods* 49 1343–1360. 10.3758/s13428-016-0790-5 27573005

[B20] Gendron M. (2017). Revisiting diversity: cultural variation reveals the constructed nature of emotion perception. *Curr. Opin. Psychol.* 17 145–150. 10.1016/j.copsyc.2017.07.014 28950961 PMC5624526

[B21] Goeleven E. Raedt R. D. Leyman L. Verschuere B. (2008). The Karolinska directed emotional faces: a validation study. *Cogn. Emot.* 22 1094–1118. 10.1080/02699930701626582 23866254

[B22] Golle J. Mast F. W. Lobmaier J. S. (2014). Something to smile about: the interrelationship between attractiveness and emotional expression. *Cogn. Emot.* 28 298–310. 10.1080/02699931.2013.817383 23875865

[B23] Heerdink M. W. van Kleef G. A. Homan A. C. Fischer A. H. (2015). Emotional expressions as social signals of rejection and acceptance: evidence from the affect misattribution paradigm. *J. Exp. Soc. Psychol.* 56 60–68. 10.1016/j.jesp.2014.09.004

[B24] Heuer K. Rinck M. Becker E. S. (2007). Avoidance of emotional facial expressions in social anxiety: the approach–avoidance task. *Behav. Res. Ther.* 45 2990–3001. 10.1016/j.brat.2007.08.010 17889827

[B25] Hong S. W. Yoon K. L. Peaco S. (2015). Sex differences in perception of invisible facial expressions. *Front. Psychol.* 6:392. 10.3389/fpsyg.2015.00392 25883583 PMC4382973

[B26] Immordino-Yang M. H. Yang X.-F. (2017). Cultural differences in the neural correlates of social–emotional feelings: an interdisciplinary, developmental perspective. *Curr. Opin. Psychol.* 17 34–40. 10.1016/j.copsyc.2017.06.008 28950970

[B27] Jusyte A. Schönenberg M. (2014). Subliminal cues bias perception of facial affect in patients with social phobia: evidence for enhanced unconscious threat processing. *Front. Hum. Neurosci.* 8:580. 10.3389/fnhum.2014.00580 25136307 PMC4120699

[B28] Kaulard K. Cunningham D. W. Bülthoff H. H. Wallraven C. (2012). The MPI facial expression database — a validated database of emotional and conversational facial expressions. *PLOS ONE* 7:e32321. 10.1371/journal.pone.0032321 22438875 PMC3305299

[B29] Lang P. J. (1980). “Behavioral treatment and bio-behavioral assessment: computer applications,” in *Technology in Mental Health Care Delivery Systems*, eds Sidowski J. B. Johnson J. H. Williams T. A. (Norwood, NJ: Ablex), 119–l37.

[B30] Lang P. J. Bradley M. M. Cuthbert B. N. (2008). *International Affective Picture System (IAPS): Affective Ratings of Pictures and Instruction Manual.* Technical Report A-8 Gainesville, FL: University of Florida.

[B31] Langner O. Dotsch R. Bijlstra G. Wigboldus D. H. J. Hawk S. T. van Knippenberg A. (2010). Presentation and validation of the radboud faces database. *Cogn. Emot.* 24 1377–1388. 10.1080/02699930903485076

[B32] Lewinski P. (2015). Automated facial coding software outperforms people in recognizing neutral faces as neutral from standardized datasets. *Front. Psychol.* 6:1386. 10.3389/fpsyg.2015.01386 26441761 PMC4565996

[B33] Lundqvist D. Flykt A. Öhman A. (1998). *The Karolinska Directed Emotional Faces - KDEF [CD ROM].* Stockholm: Karolinska Institute.

[B34] Ma D. S. Correll J. Wittenbrink B. (2015). The Chicago face database: a free stimulus set of faces and norming data. *Behav. Res. Methods* 47 1122–1135. 10.3758/s13428-014-0532-5 25582810

[B35] Manippa V. Padulo C. Brancucci A. (2017). Emotional faces influence evaluation of natural and transformed food. *Psychol. Res.* 10.1007/s00426-017-0857-7 [Epub ahead of print]. 28299462

[B36] Morrison E. R. Morris P. H. Bard K. A. (2013). The stability of facial attractiveness: is it what you’ve got or what you do with it? *J. Nonverbal Behav.* 37 59–67. 10.1007/s10919-013-0145-1 21794922

[B37] Mueser K. T. Grau B. W. Sussman S. Rosen A. J. (1984). You’re only as pretty as you feel: facial expression as a determinant of physical attractiveness. *J. Pers. Soc. Psychol.* 46 469–478. 10.1037/0022-3514.46.2.469

[B38] Murphy S. T. Zajonc R. B. (1993). Affect, cognition, and awareness: affective priming with optimal and suboptimal stimulus exposures. *J. Pers. Soc. Psychol.* 64 723–739. 10.1037//0022-3514.64.5.723 8505704

[B39] Nelson N. L. Russell J. A. (2013). Universality revisited. *Emot. Rev.* 5 8–15. 10.1177/1754073912457227

[B40] Niedenthal P. M. Rychlowska M. Wood A. (2017). Feelings and contexts: socioecological influences on the nonverbal expression of emotion. *Curr. Opin. Psychol.* 17 170–175. 10.1016/j.copsyc.2017.07.025 28950965

[B41] Paiva-Silva A. I. de Pontes M. K. Aguiar J. S. R. de Souza W. C. (2016). How do we evaluate facial emotion recognition? *Psychol. Neurosci.* 9 153–175. 10.1037/pne0000047

[B42] Paulus A. Wentura D. (2016). It depends: approach and avoidance reactions to emotional expressions are influenced by the contrast emotions presented in the task. *J. Exp. Psychol. Hum. Percept. Perform.* 42 197–212. 10.1037/xhp0000130 26322690

[B43] Penton-Voak I. S. Munafò M. R. Looi C. Y. (2017). Biased facial-emotion perception in mental health disorders: a possible target for psychological intervention? *Curr. Dir. Psychol. Sci.* 26 294–301. 10.1177/0963721417704405

[B44] Prada M. Rodrigues D. Garrido M. V. Lopes J. (2017). Food-pics-PT: portuguese validation of food images in 10 subjective evaluative dimensions. *Food Qual. Prefer.* 61 15–25. 10.1016/j.foodqual.2017.04.015

[B45] Prada M. Rodrigues D. Silva R. R. Garrido M. V. (2016). Lisbon symbol database (LSD): subjective norms for 600 symbols. *Behav. Res. Methods* 48 1370–1382. 10.3758/s13428-015-0643-7 26276520

[B46] Rodrigues D. Prada M. Gaspar R. Garrido M. V. Lopes D. (2017). Lisbon emoji and emoticon database (LEED): norms for emoji and emoticons in seven evaluative dimensions. *Behav. Res. Methods.* 10.3758/s13428-017-0878-6 [Epub ahead of print]. 28364283

[B47] Rohr M. Degner J. Wentura D. (2015). The “emotion misattribution” procedure: processing beyond good and bad under masked and unmasked presentation conditions. *Cogn. Emot.* 29 196–219. 10.1080/02699931.2014.898613 24650228

[B48] Sánchez A. Vázquez C. (2013). Prototypicality and intensity of emotional faces using an anchor-point method. *Span. J. Psychol.* 16:E7. 10.1017/sjp.2013.9 23866254

[B49] Schirmer A. Adolphs R. (2017). Emotion perception from face, voice, and touch: comparisons and convergence. *Trends Cogn. Sci.* 21 216–228. 10.1016/j.tics.2017.01.001 28173998 PMC5334135

[B50] Soares A. P. Pinheiro A. P. Costa A. Frade C. S. Comesaña M. Pureza R. (2015). Adaptation of the international affective picture system (IAPS) for european portuguese. *Behav. Res. Methods* 47 1159–1177. 10.3758/s13428-014-0535-2 25381023

[B51] Suess F. Rabovsky M. Abdel Rahman R. (2014). Perceiving emotions in neutral faces: expression processing is biased by affective person knowledge. *Soc. Cogn. Affect. Neurosci.* 10 531–536. 10.1093/scan/nsu088 24948155 PMC4381241

[B52] Tay P. K. C. Yang H. (2017). Angry faces are more resistant to forgetting than are happy faces: directed forgetting effects on the identity of emotional faces. *J. Cogn. Psychol.* 29 855–865. 10.1080/20445911.2017.1323907

[B53] Ueda R. Kuraguchi K. Ashida H. (2016). Asymmetric effect of expression intensity on evaluations of facial attractiveness. *SAGE Open* 6 1–6. 10.1177/2158244016677569

[B54] van der Schalk J. Hawk S. T. Fischer A. H. Doosje B. (2011). Moving faces, looking places: validation of the Amsterdam dynamic facial expression set (ADFES). *Emotion* 11 907–920. 10.1037/a0023853 21859206

[B55] van Dillen L. F. Harris L. T. van Dijk W. W. Rotteveel M. (2015). Looking with different eyes: the psychological meaning of categorisation goals moderates facial reactivity to facial expressions. *Cogn. Emot.* 29 1382–1400. 10.1080/02699931.2014.982514 25435404

[B56] Van Kleef G. A. van den Berg H. Heerdink M. W. (2015). The persuasive power of emotions: effects of emotional expressions on attitude formation and change. *J. Appl. Psychol.* 100 1124–1142. 10.1037/apl0000003 25402955

[B57] Wentura D. Rohr M. Degner J. (2017). Masked emotional priming: a double dissociation between direct and indirect effects reveals non-conscious processing of emotional information beyond valence. *Conscious. Cogn.* 49 203–214. 10.1016/j.concog.2017.01.016 28214770

[B58] Winkielman P. Berridge K. C. Wilbarger J. L. (2005). Unconscious affective reactions to masked happy versus angry faces influence consumption behavior and judgments of value. *Pers. Soc. Psychol. Bull.* 31 121–135. 10.1177/0146167204271309 15574667

[B59] Yan X. Andrews T. J. Jenkins R. Young A. W. (2016). Cross-cultural differences and similarities underlying other-race effects for facial identity and expression. *Q. J. Exp. Psychol.* 69 1247–1254. 10.1080/17470218.2016.1146312 26878095

[B60] Yan X. Young A. W. Andrews T. J. (2017). Differences in holistic processing do not explain cultural differences in the recognition of facial expression. *Q. J. Exp. Psychol.* 70 2445–2459. 10.1080/17470218.2016.1240816 27766927

[B61] Zebrowitz L. A. (2017). First impressions from faces. *Curr. Dir. Psychol. Sci.* 26 237–242. 10.1177/0963721416683996 28630532 PMC5473630

[B62] Zhang F. Parmley M. Wan X. Cavanagh S. (2015). Cultural differences in recognition of subdued facial expressions of emotions. *Motiv. Emot.* 39 309–319 10.1007/s11031-014-9454-x

